# In Situ Co-Amorphization of Olanzapine in the Matrix and on the Coat of Pellets

**DOI:** 10.3390/pharmaceutics14122587

**Published:** 2022-11-24

**Authors:** Nuno F. da Costa, Raquel F. Azevedo, João A. Lopes, Ana I. Fernandes, João F. Pinto

**Affiliations:** 1 iMed.ULisboa—Research Institute for Medicines, Faculdade de Farmácia, Universidade de Lisboa, Av. Prof. Gama Pinto, 1649-003 Lisboa, Portugal; 2 CiiEM—Interdisciplinary Research Center Egas Moniz, Instituto Universitário Egas Moniz, Monte de Caparica, 2829-511 Caparica, Portugal

**Keywords:** (co-)amorphous, dissolution testing, in situ (co-)amorphization, olanzapine, pellets, solubility, stability

## Abstract

In situ amorphization is a promising approach, considered in the present work, to enhance the solubility and dissolution rate of olanzapine, while minimizing the exposure of the amorphous material to the stress conditions applied during conventional processing. The production of pellets by extrusion/spheronization and the coating of inert beads were investigated as novel methods to promote the co-amorphization of olanzapine, a poorly water-soluble drug, and saccharin. Samples were characterized using differential scanning calorimetry, X-ray powder diffraction, Fourier-transform infrared spectroscopy and scanning electron microscopy, and dissolution and stability testing. The co-amorphous produced were compared with crystalline olanzapine, or physical mixture of olanzapine and saccharin. Results suggested that the addition of water to mixtures containing olanzapine and saccharin during the production of pellets, and the coating of inert beads, induced the in situ co-amorphization of these substances. The coating of inert beads enhanced the solubility and dissolution rate of olanzapine, especially when compared to pellets coated with the crystalline drug, but also with pellets containing the co-amorphous entity in the matrix of beads. Nine months stability tests (23 °C/60% RH) confirmed the preservation of the solid-state properties of the co-amorphous form on/in pellets. Overall, results highlighted the feasibility and benefits of in situ co-amorphization, either when the drug was entrapped in the pellets matrix, or preferentially applied directly on the surface of pellets.

## 1. Introduction

**Figure 1:**
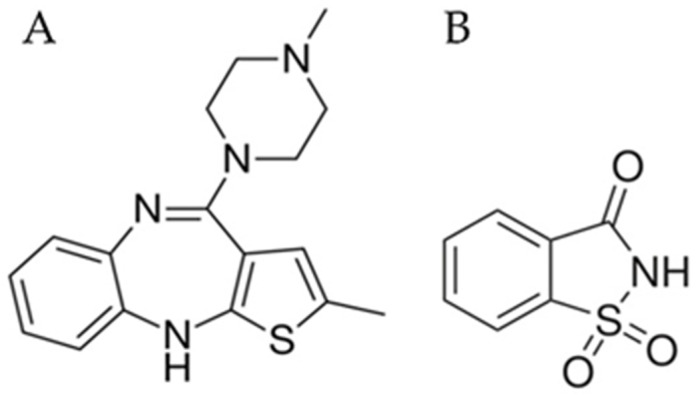
Chemical structure of olanzapine (**A**) and saccharin (**B**).

Poor water solubility of drugs, including that of many novel drug candidates, remains a major impairment on drugs’ bioavailability compromising therapeutic effectiveness [1,2]. To enhance the aqueous solubility of crystalline solids, multiple strategies are available. Among these, the formation of co-crystals has been described as a valuable strategy to enhance the water solubility and bioavailability of poorly water-soluble drugs [3,4]. However, additional strategies are warranted to further enhance the solubility of drugs and reduce the solubility concerns during the pharmaceutical development process. Disruption of the crystalline lattice, stabilization of the amorphous content, using polymers [5], or low molecular mass compounds—to produce the co-amorphous systems (CAMs) [6,7,8]—are amongst these strategies, which have been implemented with various degrees of success. Unfortunately, the high fraction of polymer commonly required to stabilize amorphous drugs (up to 90% *w*/*w*) [9] may result in the production of massive dosage forms, thus negatively impacting patient compliance [10]. In this regard, CAMs may be particularly useful for the pharmaceutical industry since higher drug loads can be used in formulations (up to 60–80% *w*/*w*) without compromising the stability of amorphous drugs [11], or the size amenable to patient administration. The formation of CAMs can be ascertained by diffractometry and calorimetry characterization methods. Diffractograms of CAMs present a characteristic halo pattern and the absence of crystalline diffraction peaks, unlike the typical diffractograms of crystalline materials (e.g., co-crystals). Additionally, thermograms of CAMs present a single glass transition temperature (T_g_), indicating miscibility of the compounds, and the absence of melting events, a feature indicative of the crystallinity of samples [12]. Olanzapine (OLZ, pK_a_ = 4.69 and 7.37 [13], Figure 1A), the model drug used in this study, is an atypical antipsychotic substance commonly administered in the treatment of schizophrenia, depression, or bipolar disorder [14]. As shown previously [15], OLZ benefits from co-amorphization with saccharin (SAC, pK_a_ = 1.6 [16], Figure 1B), both in terms of solubility (5896 vs. 41 mg/L for OLZ-CAM and pure crystalline OLZ, respectively) and dissolution rate of the drug (88.7 vs. 25.2% of drug release for OLZ-CAM and pure crystalline OLZ, respectively). Both olanzapine [17] and saccharine [18] are reportedly stable under the conditions used for co-amorphization [15]. The formation of a stable CAM between OLZ and SAC was explained by salification, rather than hydrogen or π-π bonds, which was supported by the huge difference between the pK_a_ of OLZ and SAC (∆pK_a_ = 6.06) [15]. The positive impact of the establishment of intermolecular interactions, particularly salt formation, on the physical stability of CAMs is well described in the literature [10,19,20,21,22]. Accordingly, the higher solubility and dissolution rate of OLZ in the CAM may foresee an improved bioavailability of the drug in the amorphous form, as compared to its crystalline counterpart [15].

At the industrial scale, traditional techniques used to produce amorphous and CAMs drugs include spray-drying, freeze-drying and hot-melt extrusion [1,23,24,25]. Amorphous or CAMs prepared using these techniques are often blended with pharmaceutical excipients to enhance processability during manufacture (e.g., glidants), drug release (e.g., disintegrants), acceptability/compliance (e.g., sweeteners, colorants) and handling (e.g., fillers) by the patient. Additional pharmaceutical unit operations, such as dry or wet granulation, and pelletization, are often considered to improve the flowability of materials, if required [26,27,28]. However, the stress conditions (e.g., pressure, temperature, or moisture sorption) imposed to the system during these operations may result in instability and recrystallization of the amorphous content, negatively impacting drugs’ bioavailability [29,30,31,32] and introducing product variability. Previous studies, performed by Joshi et al. [30], also reported the instability associated with the use of amorphous forms of celecoxib during compaction, resulting in the devitrification of the drug regardless of the compression pressure (between 27.4–137.5 MPa) applied to produce the compacts. These findings also hold when amorphous celecoxib was stabilized by polyvinyl pyrrolidone and meglumine, although the degree of recrystallization during compaction was substantially reduced to a value below 15% of crystallinity content [30]. Similar results were obtained by Thakral et al. [32] who prepared tablets containing amorphous indomethacin. At high compression pressures, the recrystallization of the model drug was evident immediately after the preparation of tablets [32].

Therefore, the development of strategies to induce the conversion of the crystalline into the (co-)amorphous form of drugs, and maintenance of such state, is of paramount importance. The conversion may be achieved directly during the manufacture of the dosage form, the so-called in situ amorphization, which may be advantageous to minimize the long-term stability concerns [29,33,34,35]. In addition to stabilization, in situ amorphization techniques offer the possibility to discard the production of amorphous solid dispersions prior to the manufacture of oral dosage forms, thus resulting in manufacturing savings and reduced logistic restrictions. Examples of investigations on in situ co-amorphization include carvedilol [36], furosemide, and indomethacin [33] by immersion of tablets coated with a gastro-resistant and water permeable polymer in acidic medium (0.1 M HCl). The use of radiation (e.g., microwave [37] or laser [38]) has been considered to avoid the pre-formation of the co-amorphous species prior to its downstreaming into dosage forms. Previous studies published by our research group [28,29] have reported the in situ co-amorphization of OLZ and SAC during tableting. In this respect, the extent of co-amorphization was proportional to the compression pressure and the dwell time applied to produce the compacts.

The present work aimed at the in situ co-amorphization of OLZ and SAC, present either in the matrix, or on the surface of pellets, to simultaneously enhance the OLZ solubility and dissolution rate, with the advantage of circumventing the preparation of the CAMs prior to the dosage form manufacturing process. The work also aimed at identifying the boundaries within which the in situ co-amorphization occurs.

## 2. Materials and Methods

### 2.1. Materials

OLZ (polymorphic form I, Rampex Labs Pvt. Ltd., Telangana, India), SAC (Sigma-Aldrich, Steinheim, Germany), and dichloromethane (Biochem Chemopharma, Cosne sur Loire, France) were used to prepare the OLZ-CAM. Microcrystalline cellulose (Avicel PH-101, FMC Corp., Cork, Ireland), polyvinyl pyrrolidone (K25, BASF, Ludwigshafen, Germany) and dibasic calcium phosphate anhydrous (DI-CAFOS^®^ A60, Budenheim, Budenheim, Germany), were used in the production of the pellets. Demineralized water (Destillo 2 apparatus, Herco, Freiberg am Neckar, Germany) and dichloromethane (Biochem Chemopharma, Cosne sur Loire, France) were used as granulation liquids. Hard gelatin capsules (size 0, Lonza, Basel, Switzerland) were manually filled with the pellets produced. In the preparation of the pH 8.0 phosphate buffer for the dissolution studies, sodium hydroxide (Eka Chemicals Inc., Marietta, GA, USA), and potassium phosphate monobasic (Carlo Erba Reagents, Val de Reuil, France) were used.

### 2.2. Methods

#### 2.2.1. Preparation of Powdered Co-Amorphous Olanzapine: Saccharin

OLZ-CAM was prepared by rapidly evaporating dichloromethane from a solution containing OLZ and SAC, in a 1:1 molar ratio (40 °C, 650 mbar, R-100, Buchi Rotavapor, Flawil, Switzerland). To ensure the total removal of the solvent, the product was left under vacuum for 24 h, after solvent evaporation [15]. Considering a maximum daily dose of 20 mg of OLZ, the dose of saccharin delivered to patients would be 12 mg, which is well below the acceptable human daily intake of 2.5 mg per kg of body weight [39].

#### 2.2.2. Characterization of Powdered Co-Amorphous Olanzapine: Saccharin

To ensure particle size homogeneity before further characterization, as described below, OLZ-CAM was gently milled in a mortar (to ensure maintenance of the solid state; monitored by diffractometry, calorimetry and infrared spectroscopy), and sieved through a 180 µm mesh. Preliminary studies have shown that when the same gentle process of milling imposed to the co-amorphous was applied to the respective crystalline blends of raw materials, co-amorphization of the latter was not observed.

X-ray Powder Diffraction (XRPD): XRPD measurements were conducted in a PANalytical X-ray diffractometer (X’Pert PRO, PANalytical, Almelo, The Netherlands), using a CuKα source of radiation (λ = 1.54 Å, at 40 kV and 30 mA). Analysis (approximately 500 mg of sample) were performed in the range 7–35 °2θ, at a step size of 0.017 °2θ and counting time of 50 s. Spectragryph software (Spectragryph, version 1.2.13, 2019, Oberstdorf, Germany) was used in data analysis and treatment.

Modulated Differential Scanning Calorimetry (DSC): A calorimeter (Q200, TA Instruments, New Castle, DE, USA) was used in the thermal analysis. Samples (5–10 mg) were analyzed in hermetic aluminum pans (THEPRO GbR, Heinsberg, Germany) in the −20 to 250 °C temperature range (heating rate of 5 °C/min, amplitude of 0.796 °C and 60 s period). The proprietor software (Universal Analysis 2000, version 4.7A, 2009, TA Instruments, New Castle, DE, USA) was used to analyze the thermograms. The midpoint of the change in the heat capacity baseline was taken as the T_g_ of the amorphous materials.

Fourier-transform Infrared (FTIR) Spectroscopy: a spectrometer (Alpha II, Bruker, Billerica, MA, USA) fitted with a diamond ATR accessory (Platinum ATR, Bruker, Billerica, MA, USA) was used. Samples (n = 3, approximately 50 mg) were scanned (wavenumber interval 4000–700 cm^1^, at a 4 cm^−1^ resolution), 24 times. Spectragryph software (Spectragryph, version 1.2.13, 2019, Oberstdorf, Germany) was used to analyze the data.

#### 2.2.3. Production of Pellets via Extrusion-Spheronization

Crystalline OLZ, crystalline OLZ:SAC, and OLZ-CAM were blended (10 min), with the excipients (Table 1) in a planetary mixer (Chef, Kenwood, New Lane, UK); demineralized water or dichloromethane (30% *w*/*w* on a dry basis) were used as granulation liquids. OLZ was included, at a constant fraction of 5% *w*/*w*, in both formulations I and II. After wetting of powders, materials were blended for an additional 10 min and stored in air-tight polyethylene bags for 24 h. A universal testing machine (LR50K Plus, Lloyd Instruments, Largo, FL, USA) fitted with a 50 kN load cell was used for extrusion (die length to diameter ratio of 4.0; test speed 200 mm/min). The load applied to masses was recorded as a function of the displacement of the cell using the proprietary software (Nexygen Plus, version 3.0, 2013, Largo, FL, USA). Spheronization was then conducted (1000 rpm for 20 min) on the extrudates in a radial plate spheronizer (230, Caleva, Dorset, UK). Pellets were oven-dried (40 °C) to constant mass (UM 100, Memmert, Schwabach, Germany). Pellets (200 mg, equivalent to 10 mg of OLZ) were weighted and used to fill size 0 hard gelatin capsules. Placebo pellets, without OLZ and SAC, were also produced for comparison purposes (formulation P, Table 1).

#### 2.2.4. Coating of Pellets

A fluidized bed coater (Strea 1, Aeromatic AG, Muttenz, Switzerland) equipped with a single spray nozzle was used to coat pellets using a dichloromethane or demineralized water solution/suspension of OLZ and SAC, in a 1:1 molar ratio. The drying temperature was set at 40 °C and the capacity of the fan was set at 11 units. An atomization pressure of 0.5 bar and a spay rate of 3 g/min were applied. After drying, pellets were oven dried (UM 100, Memmert, Schwabach, Germany) at 40 °C for 24 h.

#### 2.2.5. Characterization of Pellets

To gain an insight into the solid-state and solution-state properties, pellets were characterized immediately after preparation. In addition to the methods described below, XRPD, DSC, and FTIR were also used to investigate the solid-state arrangement of OLZ in samples obtained after processing and performed, as described previously (Section 2.2.2).

Drug content: physical mixtures or pellets (n = 10, 100 mg of mixtures or pellets, equivalent to 5 mg of OLZ) were placed in phosphate buffer pH 8.0 (1000 mL). After 24 h (no solid residues were visually observed), samples were diluted and drug content was estimated by using ultraviolet photometry (λ = 254 nm, U-1900, Hitachi, Tokyo, Japan).

Water content: samples (approximately 500 mg) were crushed and heated at 80 °C until constant weight. The weight variation, before and after heating, was recorded and used to determine the water content, according to Equation (1), where *W_B_* and *W_A_* represent the weight of powder before and after the drying cycle.

(1) $$\mathrm{Water}\;\mathrm{Content}\;\left(\%\right)=\frac{W_{B}-W_{A}}{W_{B}}\times 100$$

Crushing Strength: to measure the crushing strength of pellets, a texture analyzer equipped with a cylinder probe (TA.XT Plus, Stable Micro Systems, Surrey, UK) was used at a testing speed of 100 mm/min (n = 10 pellets).

Dissolution Tests: dissolution studies (paddle method, 100 rpm), were conducted in a dissolution apparatus (AT7, Sotax, Aesch, Switzerland). Approximately 200 or 2000 mg of powdered mixtures or pellets—equivalent to 10 mg (sink conditions), or 100 mg of OLZ (non-sink conditions), respectively—were placed in 1000 mL phosphate buffer pH 8.0 (to ensure that the drug is predominantly unionized), pre-heated to 37 ± 0.5 °C. At pre-defined times (0, 5, 10, 15, 30, 60, 120, 180, 240, 360, 480, 720, 960, 1200, and 1440 min), a sample of 4 mL of dissolution media was withdrawn, passed through a 0.22 µm MCE filter (Merck, Boston, MA, USA), and OLZ quantified using ultraviolet spectrophotometry, as before, to determine the fraction of OLZ released over time. Fresh dissolution medium was added to the dissolution vessel after each sample collection to maintain the dissolution volume constant throughout the test.

Scanning Electron Microscopy (SEM): the morphology of pellets was evaluated using an electron microscope (JEOL-JSM-S200LV, JOEL, Peabody, MA, USA), equipped with a secondary electron detector, at a magnification of 35×. Prior to analysis, pellets were gold coated in a sputtering chamber (JEOL JFC-1200, JOEL, Peabody, MA, USA).

Gas Chromatography (GC): the content of residual solvent (dichloromethane) was determined by injecting approximately 10 µL of sample into a gas chromatographer (Clarus^®^ 690 GC Perkin Elmer, Waltham, MA, USA) fitted with a highly sensitive ionization detector (FID).

Olanzapine solubility: approximately 300 mg of pellets (equivalent to 15 mg of OLZ) were added to eppendorfs containing 1 mL of the phosphate-buffered solution pH 8.0 pre-set at 37 °C and left to rest for 24 h (n = 3). Eppendorfs were centrifuged at 17,320× *g* for 60 min (Z 233M, Hermle, Wehingen, Germany) and filtered through a 0.22 µm MCE filter (Darmstadt, Germany). Afterwards, the filtered solution was diluted to a UV measurable concentration (𝜆 = 254 nm, U-1900, Hitachi, Tokyo, Japan), taken as the solubility of OLZ-CAM.

#### 2.2.6. Principal Component Analysis

Principal component analysis was applied to the analysis of the FTIR spectra, pre-processed using the multiplicative scatter correction method (Matlab software, R2015a, 2015, MathWorks, Sherborn, MA, USA).

#### 2.2.7. Statistical Analysis

Data analysis was conducted using the one-way ANOVA and when statistical significance was found the post hoc Tukey’s test was performed for comparison purposes (SPSS Statistics, version 27.0.1.0, 2020, IBM, New York, NY, USA). Statistical significance was considered at *p* < 0.05.

#### 2.2.8. Stability Studies

Powdered mixtures and pellets were stored in open glass vials for 9 months under room conditions (23 ± 2 °C/60 ± 5%RH) in a climatic chamber (Vötsch, VC2023, Balingen, Germany) and analyzed by XRPD to evaluate if recrystallization of OLZ-CAM had occurred.

## 3. Results

### 3.1. Differential Scanning Calorimetry and X-ray Powder Diffraction

**Figure 2:**
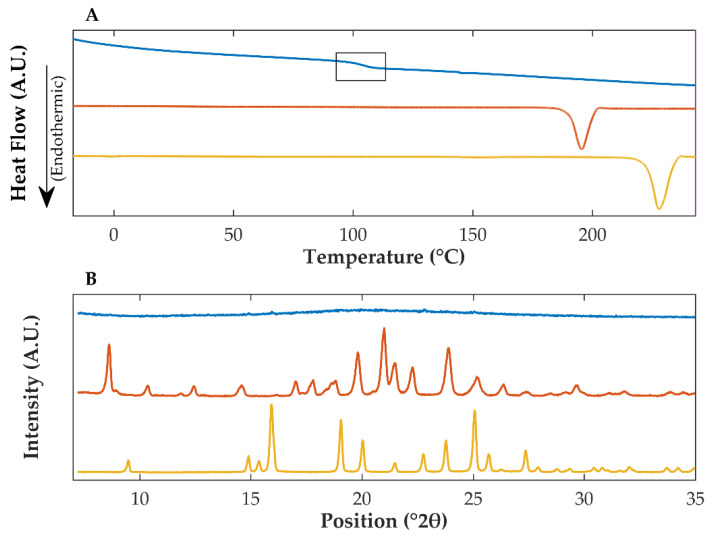
Differential scanning calorimetry thermograms (**A**) and X-ray powder diffractograms (**B**) of powdered crystalline saccharin (yellow), crystalline olanzapine (orange), and co-amorphous olanzapine:saccharin (blue). The glass transition of co-amorphous olanzapine:saccharin is highlighted in (**A**).

DSC is a thermoanalytical characterization technique applied to analyze the thermal transitions of materials (e.g., glass transitions and melting) as a function of the temperature, and hence to provide evidence of the amorphization and co-amorphization of materials [40]. The co-amorphization of a drug substance and co-amorphous stabilizers can be detected by the observation of a single T_g_ and the absence of melting events in thermograms [10]. Thermograms of pure crystalline OLZ (Figure 2A, orange) presented a single endothermic event at 192.4 °C, confirming the use of the polymorphic form I of the drug [41]. Similarly, thermograms of SAC presented a single endothermic peak at 224.8 °C (Figure 2A, yellow), in good agreement with the literature [42,43]. In contrast, the evaporation of dichloromethane from a solution containing OLZ and SAC, in a 1:1 molar ratio, resulted in a powdered sample presenting a single T_g_, at approximately 100.8 °C, and the absence of melting events (Figure 2A, blue), suggesting the co-amorphization of both compounds.

XRPD studies were run to support the aforementioned results. Diffractograms of pure crystalline OLZ presented characteristic diffraction peaks at 8.62, 19.81, 21.00, 21.08, 22.27, and 23.89 °2θ (Figure 2B, orange), comparable to diffractograms available in the literature [41] for the polymorphic form I of OLZ. Diffractograms of pure SAC presented diffraction peaks at 16.01, 19.09, 22.75, 23.86, and 25.07 °2θ (Figure 2B, yellow), also aligned with those published in the literature [7,44]. In contrast, after solvent evaporation, diffractograms of OLZ and SAC samples, presented the typical halo pattern of amorphous materials and the absence of diffraction peaks (Figure 2B, blue), which are characteristic of crystalline solids. Therefore, the combination of XRPD and DSC confirmed the co-amorphization of OLZ and SAC by evaporation of dichloromethane.

### 3.2. Fourier-Transform Infrared Spectroscopy

**Figure 3:**
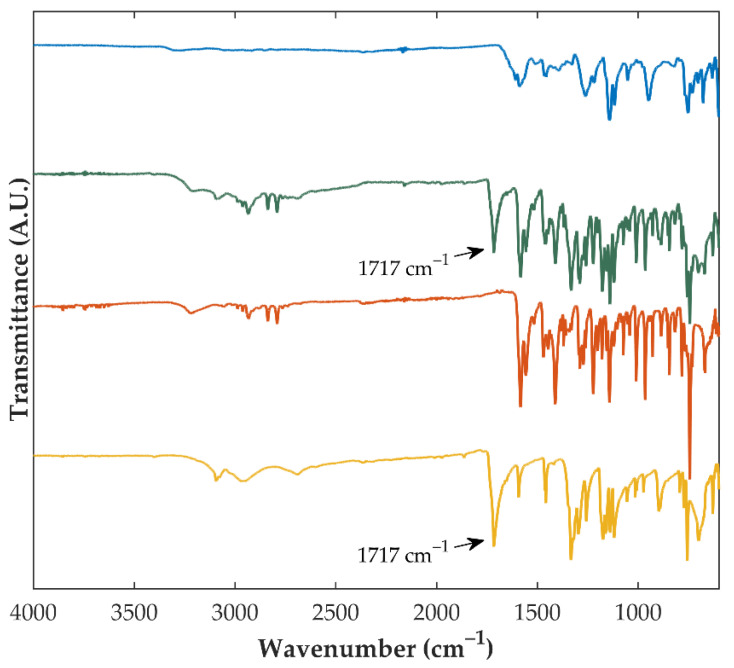
Fourier-transform infrared spectra of pure crystalline saccharin (yellow), pure crystalline olanzapine (orange), the physical mixture containing crystalline olanzapine and saccharin (green), and the co-amorphous system produced using both compounds (blue).

During the preparation of CAMs, intermolecular interactions may be established between drug(s) and co-amorphous stabilizer(s), enhancing stability of CAMs, as compared to pure amorphous drugs [45]. In this respect, FTIR spectroscopy was conducted on samples to investigate the intermolecular interactions established between OLZ and SAC. Pure crystalline OLZ presented characteristic peaks at 3218 cm^−1^ (N-H stretching), 2931 and 2791 cm^−1^ (C-H stretching), 1583 cm^−1^ (N-H stretching), 1289 cm^−1^ (C-N stretching), 965 cm^−1^ (C-S stretching) and 745 cm^−1^ (C-H out of the plan deformation) (Figure 3). Pure crystalline SAC presented peaks at 3093 cm^−1^ (N-H stretch), 1717 cm^−1^ (C=O stretch) and 1333 cm^−1^ (S=O stretching vibration). Due to the dilution of both substances, equivalent peaks were observed in the physical mixture (containing both crystalline OLZ and SAC, in a 1:1 molar ratio) spectrum but with lower intensity (Figure 3). The major difference in the spectrum of the CAM, compared to the spectrum of the respective crystalline physical mixture, was the inexistence of the C=O peak of SAC at 1717 cm^−1^, suggesting the formation of a salt (OLZ saccharinate) between the compounds, which is supported by the difference in pK_a_ between OLZ and SAC (ΔpK_a_ > 5 units) [1,10], and reported before as the mechanism of formation of these CAMs [15]. Salification is expected to promote a more stable entity than hydrogen bonds. Salt formation between OLZ and SAC is likely to prevent the recrystallization of the amorphous content, since for the recrystallization to occur, the interactions established between the compounds need to be disrupted to enable the formation and growth of crystalline nuclei [45].

### 3.3. Manufacture of Pellets Containing Olanzapine

The downstream processing of formulations containing amorphous and CAMs constitutes the last hurdle to overcome during the industrial development of novel drug products containing poorly water-soluble drugs. As discussed before, during the production of conventional oral dosage forms (e.g., tablets or pellets), stress conditions imposed on materials are likely to promote the recrystallization of the amorphous content and thus the reduction of solubility and dissolution rate. Therefore, close monitoring of the stability of CAMs during the manufacture of oral drug products is mandatory. To circumvent such problem, the in situ co-amorphization of drugs during the manufacture of pellets, or directly on the surface of inert beads during coating, is regarded as extremely advantageous. With such approach, the stress conditions to which the CAMs are exposed to are minimized, as is the risk of recrystallization. In this respect, the manufacture of pellets containing OLZ:SAC in the matrix and on the surface of inert beads was investigated in this study. For comparison purposes, pellets containing OLZ, either in the matrix or on the surface of pellets, were produced and characterized.

#### 3.3.1. Preparation of Pellets Containing Olanzapine in the Matrix

Physical mixtures were wet using dichloromethane or water, and stored for 24 h prior to extrusion. Extrusion of masses wetted with dichloromethane failed to produce extrudates possibly due to the evaporation of the solvent during storage and the high force required to extrude the masses (>50 kN). In contrast, the use of water enabled the production of extrudates which were subsequently used to produce pellets. Ranking of the force of extrusion of the formulations (P > I > II), could be related to the content in calcium phosphate. In fact, the placebo formulation P was mainly composed of anhydrous dibasic calcium phosphate (75% *w*/*w*), which is recognized by its brittle behavior during compaction. The reduction of the fraction of calcium phosphate in formulations containing OLZ, alone (71%, formulation I) or with SAC (69% *w*/*w*, formulation II), may have increased the plasticity of formulations, thus lowering the force required to extrude masses [46]. These results are in line with those of Pinto et al. [47], who have demonstrated that the use of higher ratios of microcrystalline cellulose:lactose in formulations containing indomethacin, decreased the force needed to extrude the wet masses through the die, due to the increased plasticity of formulations. Extrudates were subsequently radial plate-spheronized to produce pellets. Content uniformity analysis indicated that regardless of the formulation considered, all samples presented an average content of OLZ within the range 99.91–100.81% (*p* > 0.05, Table 2). Worth mentioning is that no individual samples contained a content of OLZ outside the limits established by the Ph. Eur. (<85 and >115%) [48], confirming the uniformity of the drug in the pellets. Additionally, the water content was below 2% (*w*/*w*) in all samples.

**Figure 4:**
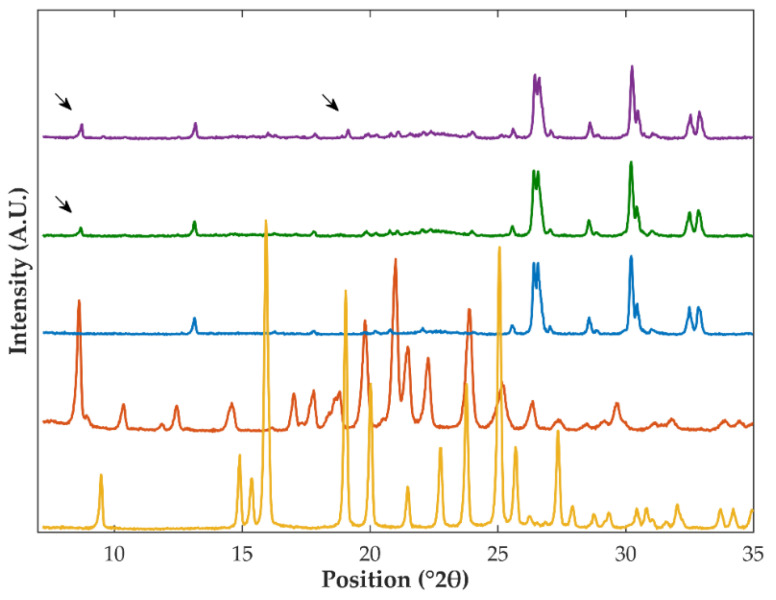
Diffractograms of crystalline saccharin (yellow), crystalline olanzapine (orange), and the physical mixtures obtained from the placebo formulation P (blue) and blends containing crystalline olanzapine (green, formulation I) or olanzapine and saccharin (purple, formulation II). Arrows highlight the peaks present in diffractograms, which could be related to either crystalline olanzapine or saccharin.

Samples were characterized using DSC and XRPD to investigate the solid-state of OLZ in extrudates and pellets. Unfortunately, DSC thermograms have not shown any thermal events in the temperature interval considered possibly due to the low fraction of OLZ in formulations (5% *w*/*w*). On the other hand, the diffractograms of the physical mixture containing crystalline OLZ or crystalline OLZ and SAC presented diffraction peaks at 8.7, 19.2, and 21.1 °2θ, which were absent in the diffractograms of the physical mixture of the placebo formulation (Figure 4). Comparison of these diffractograms with those of pure crystalline OLZ and SAC allowed association of the diffraction peaks at 8.7 and 21.1 °2θ with OLZ, whilst the peak at 19.2 °2θ was associated with the presence of crystalline SAC (Figure 4). As a result, these peaks were used as indicators of OLZ and SAC.

**Figure 5:**
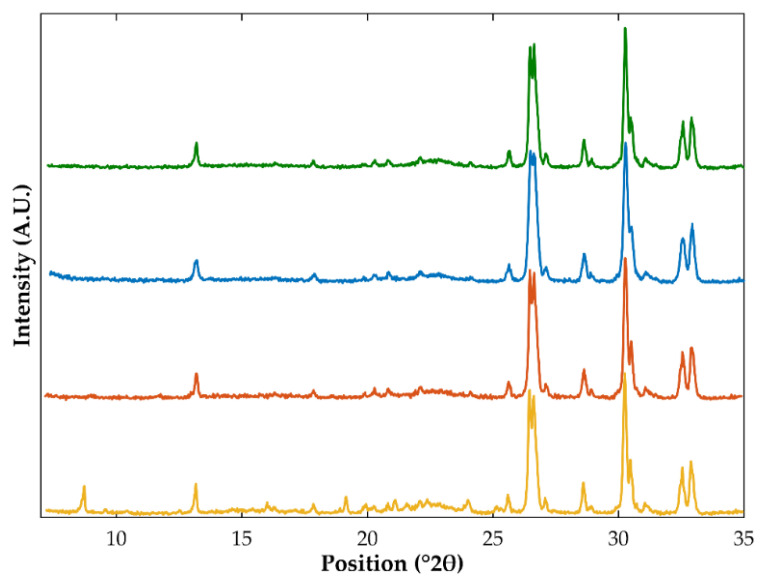
Diffractograms of samples prepared using crystalline olanzapine and saccharin (as starting material) obtained from the physical mixtures (yellow), extrudates (orange), and pellets (blue). The diffractogram of the physical mixture prepared using the co-amorphous olanzapine was plotted (green) for comparison purposes.

Extrusion and spheronization of formulation I (containing the crystalline form I of OLZ as starting material) produced samples whose diffractograms exhibited the most relevant diffraction peaks associated with OLZ (in the same position compared to the diffractograms of pure crystalline OLZ and the physical mixture prepared). Therefore, it may be assumed that no-solid state modifications of the drug have occurred during the production of extrudates and pellets (Supplementary Material—Figure S1). The same conclusions hold also true for the powder blend containing the OLZ-CAM (formulation II). Therefore, the high stability of OLZ-CAM when exposed to the different stress conditions considered in this study (e.g., solvent or drying temperature) is highlighted. In contrast, extrudates and pellets produced using formulation II (containing crystalline OLZ and SAC as starting materials) failed to show the presence of diffraction peaks related to either OLZ or SAC. These results suggest the co-amorphization of both compounds during processing (Figure 5). Water-induced co-amorphization of OLZ was previously reported by the authors [49].

To confirm the XRPD results, FTIR spectroscopy was conducted. FTIR spectra of extrudates and pellets prepared using formulation I failed to show noticeable variations, as compared to the spectrum of the physical mixture. Similar observations were made for samples containing the OLZ-CAM as starting material. Hence, FTIR spectroscopy supported the XRPD results, regarding the maintenance of the solid-state of OLZ during processing.

When samples prepared using crystalline OLZ and SAC as starting materials (formulation II) were considered, FTIR spectroscopy has shown considerable differences in the spectra of extrudates or pellets, as compared to those of the respective physical mixture. As pointed out above, the most notorious variations in the spectra of crystalline vs. OLZ-CAM were detected in the wavenumber interval 1750–1450 cm^−1^, which are likely due to the formation of a salt between both compounds (Figure 3).

**Figure 6:**
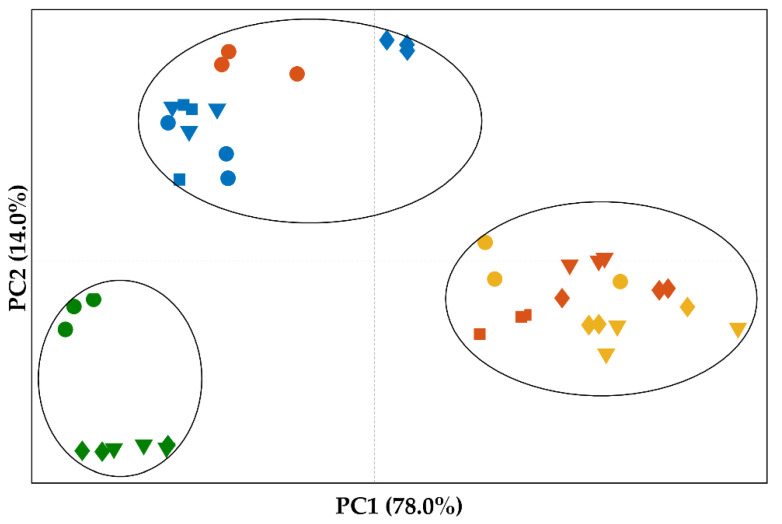
Scores plot from the principal component analysis conducted on the FTIR spectra of samples prepared using the placebo-based formulation (Formulation P, green), formulation I (blue) or formulation II, using the crystalline (orange) or the co-amorphous (yellow) form of olanzapine and saccharin, as starting material. Markers reflect the nature of samples: physical mixtures (circles), extrudates (diamonds), uncoated pellets (triangles), or pellets coated with olanzapine or olanzapine and saccharin (squares).

Principal component analysis was conducted to gain a better insight on these differences. The principal component analysis was conducted using the wavenumber interval 1750–1450 cm^−1^, where the most relevant differences were detected. The scores plot clearly shows the clustering of samples prepared using the placebo-based formulation (Figure 6, green). Similar clustering of samples was observed for samples prepared using crystalline OLZ (Formulation I, Figure 6, blue) or OLZ-CAM (Formulation II, Figure 6, yellow), as starting material. The incorporation of SAC in formulations, as a physical mixture, resulted only in minor variations in scores, in the wavenumber interval considered, as illustrated by the clustering of these samples together with those of formulations containing the crystalline form of the drug (in the absence of SAC). Inversely, the processing of this blend resulted in spectra with high similarity with formulation II containing the OLZ-CAM as starting material. In conclusion, by combining the results obtained using FTIR spectroscopy and XRPD, it may be assumed that co-amorphization of both OLZ and SAC has occurred during the production of extrudates and pellets.

Crushing strength measurements were conducted to determine the compressive load at which the structure of pellets is broken. Results have shown that the incorporation of OLZ alone in formulations (formulation I) slightly decreased the resistance of pellets (27.73 ± 1.65 vs. 23.97 ± 2.84 N for pellets produced using formulations P and I, respectively). Inversely, the addition of SAC to OLZ-containing formulations resulted in a substantial enhancement of the crushing strength of pellets (*p* < 0.01, by comparison with pellets containing OLZ in the matrix) (Table 2). These results are in line with previous publications which reported that tablets containing CAMs presented higher crushing strength values, as compared to those prepared using the respective crystalline counterparts [27,28,50]. This may be explained by the higher cohesiveness of OLZ in the CAM form [27,28,50] which may have increased the number and/or intensity of bonds established in the matrix of pellets.

**Figure 7:**
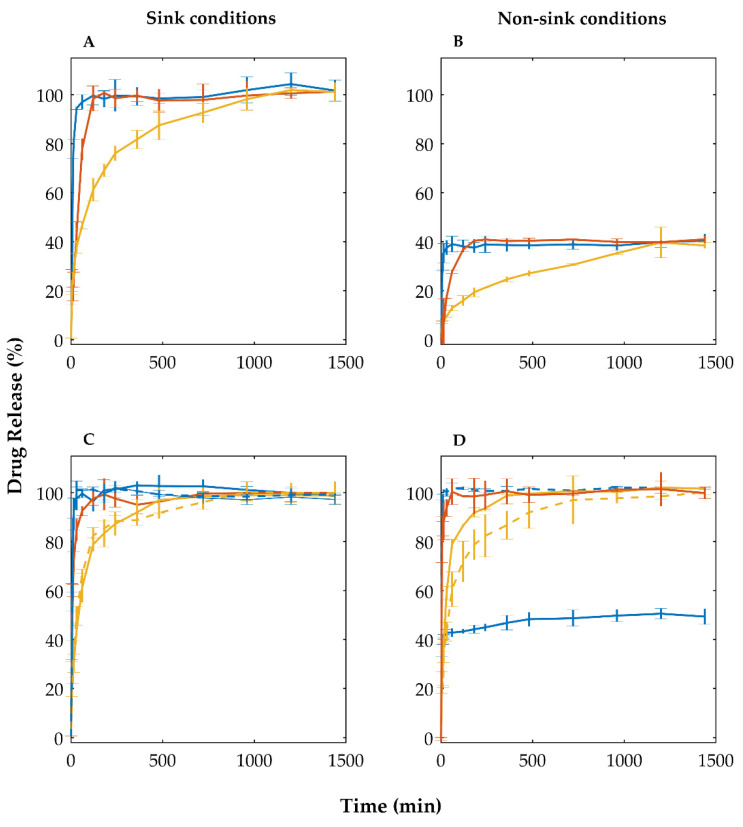
Dissolution profiles under sink (10 mg/L of OLZ, **A**,**C**) and non-sink conditions (100 mg/L of OLZ, **B**,**D**), of formulations I (**A**,**B**) and II (using the crystalline—solid line—or the co-amorphous form—dashed line—of olanzapine and saccharin, as starting materials) (**C**,**D**). Colors reflect the state of samples: physical mixtures (blue), uncoated pellets (yellow), and coated pellets (orange).

Concomitantly, dissolution studies were conducted, under sink and non-sink conditions (to reflect the poor solubility of crystalline OLZ, by contrast to amorphous OLZ), to evaluate the rate and extent of OLZ release. Dissolution profiles of samples, under sink conditions (10 mg/L of OLZ, equivalent to 200 mg of pellets), indicated that all samples released the entire quantity of the drug into the dissolution media after 24 h of test, as expected. Particularly, pellets containing OLZ in the matrix (formulation I) resulted in dissolution profiles presenting a slow release of the drug from the structure of pellets (Figure 7, yellow). The dissolution rate of OLZ from samples was considerably enhanced by introducing SAC in formulations, as reflected by the reduction of the time required to release 75% of the drug (89 vs. 253 min for pellets containing crystalline OLZ:SAC and crystalline OLZ, respectively; *p* < 0.01; Table 2). Interestingly, pellets of formulation II containing OLZ-CAM in the matrix exhibited a slightly slower release of the drug when compared to pellets prepared using the crystalline OLZ:SAC as starting material (t_50_ of 39 and 31 min for pellets containing OLZ-CAM and crystalline OLZ:SAC; *p* < 0.05; Table 2). The slower release of the drug in the CAM from pelletized samples may be related to the higher crushing strength of pellets containing OLZ-CAM (Table 2); the penetration of water into the matrix of the beads may have been hampered by the more compact structure, thus slowing down the release of the drug. Nevertheless, pellets containing OLZ-CAM in the matrix have shown to significantly enhance the drug dissolution rate by comparison with pellets without the co-amorphous stabilizer (SAC, Table 2, *p* < 0.01). Inversely, dissolution tests conducted under non-sink conditions (100 mg/L of OLZ, equivalent to 2 g of pellets) on formulation I-based samples have shown the stabilization of the release of the drug at approximately 40%, in line with the solubility of the crystalline drug in the dissolution media (41 mg/L, at 37 °C) which guaranteed the maintenance of the non-sink conditions throughout the test. Both the co-amorphization of OLZ and SAC, by solvent evaporation (prior to the manufacture of pellets) or in situ (during the production of pellets), increased the equilibrium concentration of the drug. Therefore, based on the higher dissolution rate and equilibrium concentration of OLZ-CAM in pellets, one may expect enhanced oral bioavailability of the drug and maximization of the therapeutic effectiveness of the treatment. Noteworthy is that the dissolution profiles of studies performed at pH > 8.0 did not show significant differences. At lower pH, the solubility of the drug, even in the absence of SAC, was significantly increased and so was release rate and dissolution, thus not guaranteeing the required non-sink conditions.

#### 3.3.2. Preparation and Characterization of Pellets Containing Olanzapine on the Surface

**Figure 8:**
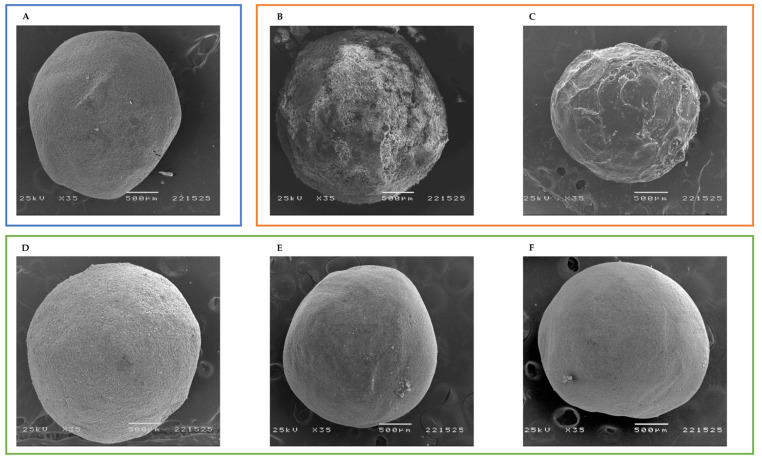
Scanning electron microphotographs of placebo-based pellets (**A**), pellets containing olanzapine (**B**) or olanzapine and saccharin (**C**) on the surface and pellets containing olanzapine in the matrix of the bead—formulation I (**D**) and formulation II, using olanzapine and saccharin in the crystalline (**E**) or the co-amorphous (**F**) form, as starting materials.

After the manufacture of placebo-based pellets, beads were film-coated using solutions of dichloromethane and OLZ or OLZ:SAC, or water suspensions of OLZ or OLZ:SAC. Unfortunately, spraying of water suspensions, clogged the atomization nozzle and the pellets produced presented an OLZ content of approximately 54% (equivalent to 2.7% *w*/*w* of OLZ in pellets), well below the drug content of OLZ aimed. On the contrary, the use of solutions of OLZ, or OLZ and SAC, in dichloromethane, resulted in the manufacture of pellets presenting the desired content of the drug (100.48 ± 8.20% or 100.81 ± 7.92%, respectively). SEM microphotographs (Figure 8) indicated that the coating of placebo-based pellets, using a solution of OLZ or OLZ:SAC, resulted in pellets presenting a rough surface (Figure 8B,C) with an outer layer of approximately 20 µm thick (readers are recommended to the Supplementary Material to observe the cross section of pellets; Figure S2). In contrast, pellets containing the model drug in the matrix presented a clean and smooth surface (Figure 8D–F). Since dichloromethane may result in safety concerns for patients, gas chromatography analysis was conducted on coated pellets to determine the concentration of the solvent. Results have shown that for all samples dichloromethane was present in pellets at a concentration lower than 200 ppm, thus well below the maximum concentration of residual solvents imposed by the ICH Q3C (<600 ppm) [51]. Furthermore, considering the maximum daily intake of OLZ (20 mg), the quantity of solvent administered to the patients lies well below the permissible daily exposure established by the guideline (<6 mg/day) [51].

As before, the solid-state arrangement of OLZ in samples was investigated using XRPD and FTIR spectroscopy. Diffractograms of samples obtained from pellets coated using the dichloromethane OLZ solution, presented the diffraction peaks of OLZ at 8.7 and 21.1 °2θ (Supplementary Material—Figure S1), in line with the polymorphic form I of the drug (as discussed in Section 3.3.1). Likewise, the FTIR spectra of samples obtained from pellets containing OLZ on the surface did not show any noticeable modifications, when compared to those of the physical mixture and pellets containing OLZ in the matrix. Therefore, one may conclude that OLZ recrystallized during the coating of inert beads.

On the other hand, the incorporation of SAC into the spraying solution resulted in the production of coated pellets, whose diffractograms failed to present the diffraction peaks of both OLZ and SAC. Worth highlighting here, that no additional peaks were observed in diffractograms and those present therein were related to the excipients used to produce the pellets. Furthermore, the FTIR spectra of pellets coated with OLZ and SAC did not present the peak characteristic of the C=O group of SAC (at approximately 1717 cm^−1^), suggesting the interaction of OLZ and SAC in the solid-state. Consequently, co-amorphization of OLZ and SAC is suggested by the results of both techniques.

Multivariate analysis (Figure 6) allowed identification of clustering of samples obtained from pellets coated with OLZ and SAC with those prepared using the OLZ-CAM, previously prepared by solvent evaporation, as starting material. The high similarity of the spectra of both samples was thus ascertained. Consequently, FTIR spectroscopy reinforces the co-amorphization of OLZ and SAC during the coating of inert beads. In fact, the low boiling temperature of dichloromethane (40 °C, [52]) and the atomization of solutions into small droplets may have resulted in a rapid evaporation of the solvent and hence a short time available for the arrangement of molecules.

Measurements of the crushing strength of pellets have shown that the coating of beads using solutions containing OLZ or OLZ:SAC had no significant impact on the internal resistance of the pellets as compared to the values obtained for placebo-based pellets and pellets containing OLZ in the matrix (*p* > 0.05, Table 2). These results are expected since the pellets coated by either OLZ or OLZ:SAC presented the same common matrix (placebo-based pellets). In contrast, the internal resistance of the pellets coated with OLZ or OLZ:SAC was significantly reduced by comparison with the pellets containing OLZ:SAC in the matrix (Table 2).

Deposition of OLZ or OLZ:SAC on the surface of pellets considerably enhanced the dissolution rate of the drug as compared to samples containing OLZ in the matrix (*p* < 0.01). Dissolution of pellets containing OLZ on the surface has shown that at 1 h of test more than 75% of the drug was already dissolved in the dissolution media (Table 2). This represents a 4-fold enhancement in the drug dissolution rate compared to samples containing OLZ in the matrix. Dissolution tests, under non-sink conditions (100 mg/L of OLZ, equivalent to 2 g of pellets), showed the stabilization of the drug release at approximately 40%, equivalent to 40 mg/L, aligned with the solubility of the polymorphic form I of the drug, as discussed before.

Pellets containing OLZ and SAC on the surface of beads released the drug faster—approximately 75% of the drug in 14 min, which stands for a 6- and 7-fold enhancement compared to pellets containing OLZ:SAC or the CAM, as starting materials, in the matrix of pellets, respectively. The higher dissolution rate of OLZ from the surface of pellets was also observed in dissolution tests conducted under non-sink conditions (Figure 7D). Under non-sink conditions, it was also shown that the pellets coated with OLZ:SAC in dichloromethane, released the total amount of OLZ inserted in the dissolution vessel (100 mg of OLZ/L). In fact, the solubility of OLZ-CAM in pellets has shown to be considerably enhanced to values (5897.5 ± 194.9 mg/L for pellets containing OLZ:SAC on the surface, and 5903.3 ± 279.1 and 5847.9 ± 281.7 mg/L for pellets containing OLZ:SAC and OLZ:CAM in the matrix, respectively) way above that of the crystalline counterpart. It is worth highlighting that no statistical differences were found for the solubility of OLZ from pellets containing OLZ:SAC on the surface, or pellets containing OLZ:SAC and OLZ:CAM in the matrix (*p* > 0.05). The results obtained stand for about a 145-fold enhancement in the solubility of OLZ and are aligned with previous works [15].

These results highlighted the value of coating as a novel preparation technique to generate CAMs of poorly water soluble drugs directly during the production of dosage forms. Compared to conventional manufacture methods using CAMs, this technique precludes the prior preparation of the CAMs, thus reducing the number of unit operations required in the manufacturing process and the associated costs; stability concerns, such as the recrystallization of the amorphous content due to the exposure to stress conditions (e.g., temperature or humidity), are also minimized. In addition, coating of inert beads has proven to considerably enhance the solubility and dissolution rate of the drug from pellets, as compared to conventional pellets in which drugs are present in the matrix, thus anticipating a higher oral bioavailability.

### 3.4. Evaluation of the Stability of Co-Amorphous Olanzapine in Pellets

**Figure 9:**
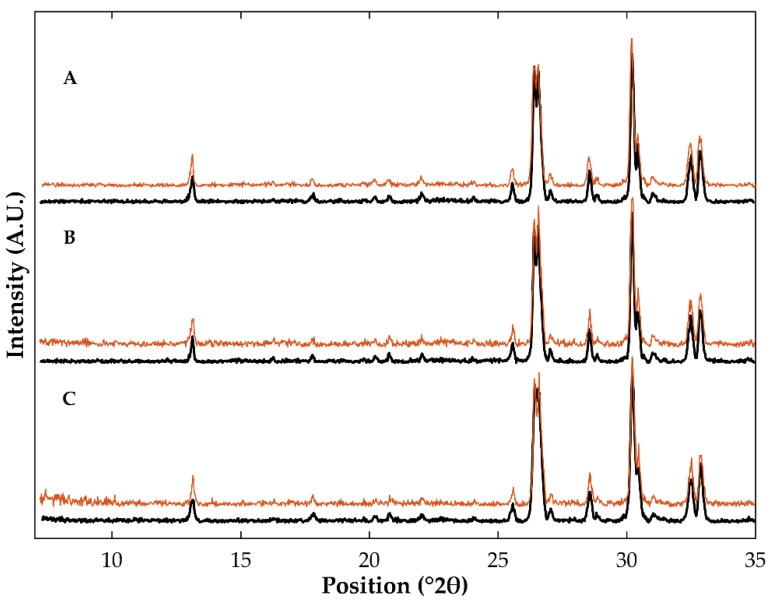
Diffractograms of samples obtained from pellets containing olanzapine and saccharin on the surface (**A**) or in the matrix of the beads, using the crystalline (**B**) or the co-amorphous form (**C**), as starting materials. Colors reflect the storage time: 0 (black) or 9 months (orange).

Stability studies were run for 9 months at 23 °C/60% of relative humidity (shelf storage conditions) to evaluate the extent of recrystallization of OLZ-CAM in pellets. At the end of the stability test, diffractograms of samples (Figure 9) did not show any noticeable variations, when compared to diffractograms of recently manufactured samples, suggesting the maintenance of OLZ in the co-amorphous form during the storage time considered. The formation of OLZ saccharinate by salification may have prevented the reorganization of molecules, explaining the high stability of the OLZ-CAM. These results are aligned with previous studies, which reported that the CAM containing OLZ and SAC was stable for more than 3 years, at 23 °C/65% RH [28]. The storage of samples for longer periods of time is recommended to compare the rate of recrystallization of OLZ-CAM from samples containing the drug in the matrix or on the surface of pellets.

## 4. Conclusions

The production of pellets containing OLZ in the highly soluble co-amorphous form, either in the matrix, or on the surface of pellets, was achieved. The stability of OLZ-CAMs throughout the manufacture of pellets was confirmed, since no signs of drug recrystallization were detected. Water-induced in situ co-amorphization of crystalline OLZ and SAC, during the production of pellets, resulted in solubility increase and a faster release of the drug from the matrix (t_75%_ < 89 min vs. t_75%_ > 252 min, for pellets containing crystalline OLZ in the matrix). Conversely, coating of inert beads, using an organic solution of OLZ and SAC (1:1 molar ratio), was established as a novel method to prepare CAMs in situ, both enhancing the solubility and the dissolution rate (t_75%_ < 14 min) of the drug. Compared to the conventional methods of pellet production from CAMs, the co-amorphization of OLZ achieved directly on the surface of beads, not only minimizes the recrystallization risk but also is likely to reduce the production costs and time, since no previous production of CAMs is needed. The added-value of the coating-induced co-amorphization, as a novel preparation technique to generate CAMs, was confirmed in this work. In the future, the same approach should be extended to other poorly water-soluble drugs, combinations of drugs and co-amorphous stabilizers (e.g., amino acids) and considered in tailoring drug release, once long-term stability has been proved for such new entities.

## Figures and Tables

**Table 1:** Composition of formulations (% *w*/*w*) used in the study.

	Formulation		
Material	P	I	II *
OLZ	-	5	5
SAC	-	-	3
Dibasic calcium phosphate anhydrous	75	71	69
Microcrystalline cellulose	20	19	18
Polyvinylpyrrolidone	5	5	5

* the proportion of 5 OLZ: 3 SAC represents a 1:1 molar ratio.

**Table 2:** Properties of pellets containing OLZ, or OLZ and SAC, in the matrix or on the surface.

	Matrix			Surface	
	OLZ	OLZ:SAC	CAM	OLZ	OLZ:SAC
OLZ content (% *w*/*w*)	100.19 ± 4.71	100.02 ± 6.79	99.91 ± 2.22	100.48 ± 8.20	100.81 ± 7.92
Crushing Strength (N)	23.97 ± 2.84 **	33.61 ± 2.44 ^##^	36.38 ± 2.02 ^##^	23.76 ± 3.18 **	24.67 ± 2.47 **
t_50%_ (min) ^1^	70.71 ± 3.35 **	31.36 ± 4.28 ^##^	39.25 ± 3.90 ^##,^*	35.37 ± 3.30 ^##^	6.72 ± 1.78 ^##,^**
t_75%_ (min) ^1^	252.86 ± 22.99 **	88.77 ± 8.95 ^##^	102.17 ± 10.04 ^##^	58.89 ± 4.29 ^##,^*	13.96 ± 1.15 ^##,^**

^1^ t_50_ and t_75_ represent the time required to achieve a drug release of 50 and 75%, respectively (under sink conditions, 10 mg/L of OLZ); ^##^ *p* < 0.01 vs. pellets containing OLZ in the matrix (crystalline OLZ, as starting material); * *p* < 0.05 and ** *p* < 0.01 vs. pellets containing OLZ and SAC in the matrix (crystalline OLZ and SAC, as starting material).

## Data Availability

All data generated or analyzed during this study is included in this published article [and its Supplementary Information Files].

## Supplementary Materials

The following supporting information can be downloaded at: https://www.mdpi.com/article/10.3390/pharmaceutics14122587/s1, Figure S1: Diffractograms of samples obtained from placebo formulations (**A**), and formulations containing olanzapine (**B**) or olanzapine:saccharin in the crystalline (**C**) or the co-amorphous (**D**) form, as starting materials; Figure S2: Scanning electron microphotographs of the cross sectional area of pellets containing olanzapine (formulation I, **A**) or olanzapine and saccharin (formulation II, **B**) on the surface of the bead.
