# Supplementary material for: In Situ Co-Amorphization of Olanzapine in the Matrix and on the Coat of Pellets
Source: Pharmaceutics. 2022 Nov 24;14(12):2587. doi: 10.3390/pharmaceutics14122587 (PMC9783598; doi:10.3390/pharmaceutics14122587)
Supplement: Supplementary file 1 [file pharmaceutics-14-02587-s001.zip › pharmaceutics-2007325-supplementary.pdf]

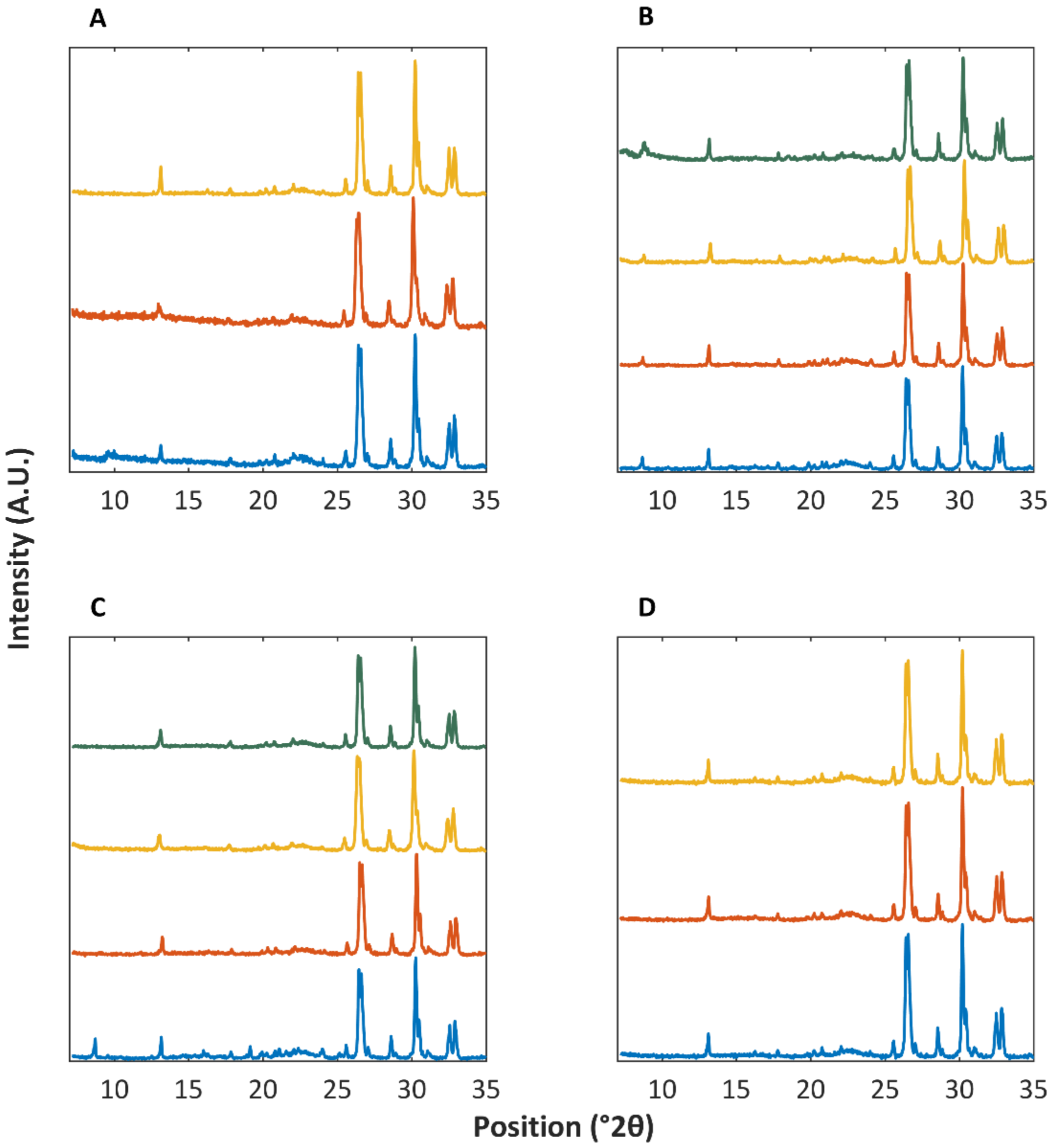

Figure S1. Diffractograms of samples obtained from placebo formulations (**A**), and formulations containing olanzapine (**B**) or olanzapine:saccharin in the crystalline (**C**) or the co-amorphous (**D**) form, as starting materials. Colors reflect the nature of samples: physical mixture (blue), extrudate (orange), pellets (yellow) and pellets coated with olanzapine or olanzapine and saccharin (green).

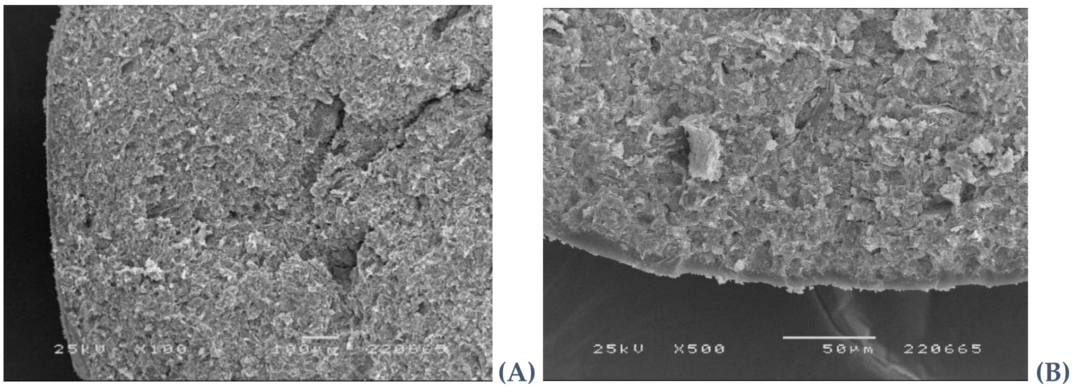

(A) **(B)**

Figure S2. Scanning electron microphotographs of the cross sectional area of pellets containing olanzapine (formulation I, **A**) or olanzapine and saccharin (formulation II, **B**) on the surface of the bead.
